# Technology Integration to Support Nurses in an “Inpatient Room of the Future”: Qualitative Analysis

**DOI:** 10.2196/68689

**Published:** 2025-06-16

**Authors:** Elizabeth R Stevens, Veronica Alfaro Arias, Son Luu, Katharine Lawrence, Lisa Groom

**Affiliations:** 1 Department of Population Health NYU Grossman School of Medicine New York, NY United States; 2 Department of Health Informatics Medical Center Information Technology NYU Langone Health New York, NY United States

**Keywords:** nursing clinical workflows, patient room design, user-centered design, health information technology, digital health, built environment, technology integration, human-centered design

## Abstract

**Background:**

The design and integration of technology within inpatient hospital rooms has a critical role in supporting nursing workflows, enhancing provider experience, and improving patient care. As health care technology evolves, there is a need to design “future-proofed” physical environments that integrate technology in ways that support workflows and maintain clinical performance. Assessing how current technologies affect nursing workflows can help inform the development of these future environments.

**Objective:**

We assessed the current challenges nursing staff face in inpatient rooms, gather insights on technology, and build environment interactions to envision the design of a technology-integrated “Inpatient Room of the Future.”

**Methods:**

A qualitative study was conducted involving semistructured interviews, shadowing, and focus groups among nursing staff in the inpatient setting. Methods including horizon scanning, scenario analysis, technology assessment, and backcasting facilitated a comprehensive qualitative analysis of current technology use and needs in inpatient nursing workflows to inform exploratory design considerations for technology-integrated envisioned futures solutions.

**Results:**

In total, 26 nursing staff across 4 inpatient hospital units participated in this study. Analysis identified four major themes considered central to designing a technology-integrated inpatient room that enhances nursing workflow and experience: (1) the need for seamless integration of technologies advocating for a unified system that minimizes fragmented technology use and enhances efficiency; (2) the potential for technology to reduce cognitive load, alleviate mental strain, and streamline complex workflows; (3) a focus on enhancing interpersonal communication with specific emphasis on tools that facilitate clear and efficient communication among clinicians and with patients; and (4) the importance of improved staff well-being with design considerations aimed at promoting both physical and mental health for health care workers in the inpatient setting. Envisioned future solutions included enhanced patient monitoring with automated measurements and actions through computer vision and data triangulation, a smart electronic health record–integrated supply management system using computer vision to detect supply shortages and auto-delivery of needed supplies, and a personal tech smart assistant capable of real-time patient monitoring and escalation, task prioritization, and hands-free clinical documentation and communication.

**Conclusions:**

While current technologies address specific tasks, there are significant opportunities for better technology integration, reducing cognitive load, enhancing communication, and promoting the physical and mental well-being of nursing staff. Future research should focus on seamless technology integration aligned with clinical workflows and implementing supportive technologies that do not interfere with clinician judgment and critical thinking. Policy recommendations include oversight mechanisms for evaluating artificial intelligence–enabled devices, safeguarding patient information, and ensuring nurses are actively involved at every stage of technology development and implementation. Future inpatient unit designs should actively engage input from both nursing professionals and technologists in developing future-proofed clinical spaces to ensure the creation of integrated systems that foster a cohesive and harmonious user experience.

## Introduction

The design of an inpatient hospital room, including both its physical layout and the technology within it, significantly impacts health care provider experiences and patient outcomes [1,2]. Room design can help patients feel safe, promote a sense of calm, improve care satisfaction, and reduce length of stay [3,4]. Thoughtfully designed inpatient rooms are also linked to enhanced clinician satisfaction, increased market share, and decreased staff turnover, while poor design can disrupt workflows and hinder care delivery [5-7]. Critically, the design of hospital spaces, including streamlined nursing stations, single-bedded rooms, and improved line of sight to patient care spaces, is linked to health care outcomes, contributing to an evidence base in support of designing the built environment to improve patient care [8,9].

Nursing staff are uniquely affected by the design of clinical rooms and the technology within them. Physically demanding tasks such as medication administration and patient repositioning require efficiency in room design to minimize unnecessary movement and physical strain [10]. Factors such as patient-to-nurse ratios, assistance requests via nurse call systems, and alarm messaging add complexity to nursing routines, high patient volumes often limit nurses’ time with individual patients, and time-intensive care for complex patients shorten windows to complete routine tasks such as medication administration [11,12].

Against this backdrop, the introduction of emerging health technologies meant to support care delivery risks creating an environment of cognitive strain, context switching, and distractions [6], all of which increase risks to patient safety and jeopardize health care outcomes. For example, centralized situation displays (eg, monitors that aggregate and show patient vital data and other parameters) can improve information access and speed of work, but may reduce bedside care and time spent with patients [5,13]. Hands-free communication and alert devices can enhance safety by notifying nurses of patient needs or changing conditions, though poorly implemented alarms may lead to “alarm fatigue,” “confusion,” and “safety risks” [14]. Finally, the physical layout and mobility of electronic health record (EHR) documentation locations (eg, “touchdown” stations) can either facilitate or impede important patient communication and education [15].

The thoughtful integration of emerging technologies in clinical spaces is vital for developing “future-proofed” environments that optimize nursing care delivery. A critical step in this work is understanding how technology is currently used by nursing staff—including physical and digital workflows, pain points, and opportunities areas—and future-casting where emergent technologies such as nursing robotics [16], ambient documentation, and computer vision might align with the built environment to optimize their work. While existing literature examines patient room design and emerging technologies [17,18], research rarely takes a future-oriented approach to evaluating the intersection of nursing, technology, and the built environment and their implications for “future-proofing” care delivery. There is a lack of studies that move beyond identifying current challenges to envisioning future scenarios and solutions, limiting the application of futures methodologies in nursing and health care design.

To address this gap, we sought to delineate key considerations for the integration of emergent technologies into inpatient nursing care by answering the questions of how do inpatient nursing staff (1) use current technologies in their work, (2) experience challenges related to these technologies, and (3) articulate preferences and priorities regarding future technologies. By engaging with nursing staff working in the inpatient setting through clinical shadowing, semistructured interviews, and focus groups (FGs), this work offers key insights to guide the design of future-facing, technology-integrated clinical environments that optimize nursing care.

## Methods

### Overview

This study was conducted as part of a medical futures initiative aimed at envisioning the future of technology-enabled care delivery and ensuring the long-term adaptability of a large urban health care institution. Led by an innovation laboratory within the health care system’s IT department, the initiative focuses on exploring, designing, and testing potential health care solutions for emerging technologies. The laboratory uses a variety of human-centered design (HCD) methodologies to conceptualize, design, and develop new innovations, including traditional qualitative design and design research approaches [19], experience design [20], rapid prototyping and prototype testing, and medical futures studies [21].

In this study, a future-oriented HCD framework was applied to move beyond problem identification toward envisioning potential future scenarios and solutions [22,23]. These approaches included ethnographic futures research [24], which combines traditional ethnography with future-oriented scenario building to assess how nursing workflows and technology use might evolve, and design futures [25], which integrates speculative design with participatory methods to cocreate visions of future clinical environments.

### Setting and Participants

The study took place across four American Nurses Credentialing Center Magnet–designated hospitals in a private, urban, nonprofit health care system covering New York, New Jersey, Connecticut, and Florida, representing over 1200 inpatient beds and multiple clinical specialties. Participants included registered nurses (RNs) and Patient Unit Associates (PUAs) from these hospitals, who were selected due to their continuous presence in inpatient rooms and direct involvement in patient care. Recruitment was conducted via convenience sampling, with inpatient unit managers reaching out to eligible participants. Organizational nursing leadership and the nursing informatics department provided recommendations of specific nursing units to engage, due to the availability and use of technologies existing in those units. All recruitment occurred through email. RNs were specifically chosen as the target population as they represent a category of staff heavily impacted by room design and technology.

### Data Collection and Analysis

**Figure 1:**
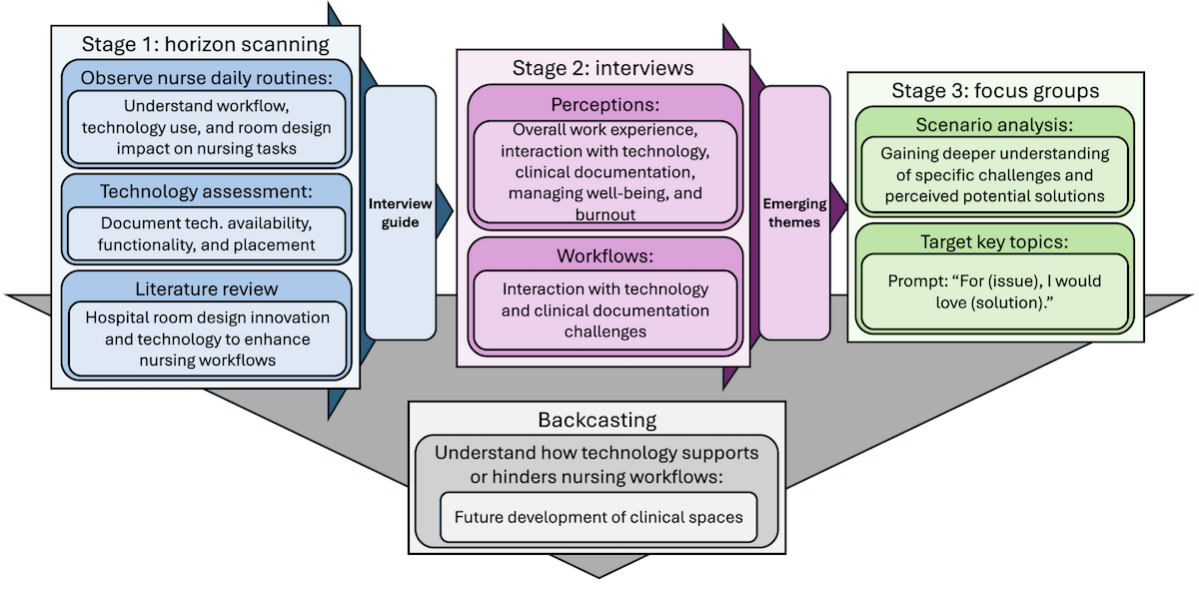
Study phases and methods workflow diagram.

This initiative was grounded in health futures methodology and used medical futures foresight methods [26]. Before the qualitative research, horizon scanning, including a search of relevant nursing and futures literature, was conducted to identify emerging technologies (eg, computer vision, voice technology, robotics, ambient room sensing, and biometric sensors) for inpatient care and previously identified nursing-specific technology considerations. Experts in nursing informatics were also consulted during study development to inform technology assessments and guided interviews, and FG discussions with nurses. Data collection was organized into 3 iterative phases: clinical shadowing, semistructured interviews, and FGs. Data collection guides for each study phase were iteratively developed using data collected from the previous study phase (ie, the interview guide was developed using horizon scanning and shadowing results, while the FG guides were developed using horizon scanning, shadowing, and interview results). The iterative inclusion of study results into the development of data collection guides was grounded in an HCD design approach framework that focuses on the needs and experiences of users to create effective solutions and incorporates concepts of horizon scanning, technology assessments, and future scenario analysis [15,21]. A summary of the study phases and methods is depicted in Figure 1.

Shadowing involved multiple 1- to 3-hour sessions to observe nurses in their daily routines to understand workflow, technology usage, and the impact of room design on nursing tasks. Research staff members (VAA and SL) were trained to document RN behaviors and technology used, and to probe with follow-up questions to explain observed behaviors, identify perceived challenges, and explore opportunities for improvement. During these sessions, researchers also conducted a technology assessment to document the availability, functionality, and placement of technologies within units. Observations were recorded in field notes and subsequently imported into Figma (Figma Inc) [27], a user experience design platform supporting multimedia data for qualitative data analyses.

Following shadowing, the interview guide was developed to incorporate insights from horizon scanning, nurse shadowing, the technology assessment, and a literature review on hospital room design, design innovations, and technology use to enhance nursing workflows. To gain a deeper understanding of observations made during horizon scanning and shadowing, interview topics included prompts to elicit nurses’ perceptions of their overall work experience, workflow interactions with technology use, clinical documentation workflows and challenges, and experiences managing well-being and preventing burnout. Researchers conducted 30- to 45-minute semistructured interviews (in-person or via videoconference) with individual nurses. To protect participants’ privacy, interviews were neither audio- nor video-recorded. Instead, each interviewer took handwritten shorthand notes during the sessions, which were then expanded and detailed immediately after each interview to ensure accuracy and completeness. To mitigate bias and maintain consistency in the data collection, research staff were trained in interviewing techniques and note-taking, using templates designed for each research activity.

After shadowing and in-depth interviews were completed, emerging themes were compiled and assessed via FGs. To affirm the previous observations and gain a deeper understanding of the specific challenges faced by inpatient RNs, along with their envisioned potential futures solutions, FG participants were instructed by research staff (VAA and SL) to identify any issues pertaining to a predefined list of broad topic category prompts and suggest a potential solution to that issue in the format of writing: “For [issue], I would love [solution].” The prompt topic categories were generated using the literature and results from previously performed shadowing and interviews. The prompt category list included (1) physical and emotional stress, (2) ease of device use, (3) physical constraints, (4) clinical documentation, (5) physical care of patients (turning and wound care), (6) patient education, (7) interruptions, (8) task management and prioritization, (9) vital sign monitoring, (10) alarm management, (11) multiple devices and software systems for routine tasks, (12) communication and handoff, (13) room supplies and turnover, and (14) technology and device support. After creating their written responses to the prompts, the RNs were allowed to verbally explain and add to what they had written.

Using futures scenario analysis, FG nurses were also engaged in speculative design activities to explore the potential impacts of these technologies. Nurses were given cards that described specific future technology solutions (Figure 1). They were asked to describe their thoughts on these technologies and provide ideas for what else they wished the technology to do. Additionally, they were asked whether the technology would improve their day-to-day work and to brainstorm how these innovations could interact with each other. Verbal discussions were recorded via field notes, and handwritten prompt responses were collected and transcribed into a digital format. Each FG occurred in person, included 2-3 nurses, and lasted approximately 30 minutes.

Interview, shadowing, and FG notes were coded using procedures designed to ensure thoroughness and reliability. Data were coded according to the principles of the “framework for applied research” [28], which consists of a 5-stage process including familiarization, identifying themes, indexing, charting, and interpretation. Codes were primarily developed a priori based on the quality improvement goals of the study and designed to capture elements such as, but not limited to, room features (eg, physical layout, technology, and noise), technology aspects (eg, integration and barriers to use), and nurse workflow (eg, tasks, decision points, communication, and interaction with technology or space). Additional codes were developed by reviewing, group coding, and discussions of a random sample of interviews as a team. The general development of themes arose from the data, using the principles of grounded theory [29]. While coded data were combined for thematic analysis, participants were given identification numbers associated with their role and the activity in which they participated, including shadowing nurse (SN), shadowing PUA, FG, and interview nurse (IN). To enhance reliability, two researchers (VAA and SL) conjointly took part in the coding and analysis process for each interview, and any disagreements were discussed as a group. Due to the joint nature of the coding process, intercoder reliability was not calculated. When coding was complete, the outputs of quotations with each code were examined, summarized, and grouped into themes. As a last step, backcasting was used to cocreate a desired future for inpatient care and to identify steps to achieve it.

### Ethical Considerations

This was an observational study of a quality improvement initiative. At the outset of this work, we anticipated that participants (nurses) would be engaged in the design process and would be solicited for feedback, which would inform prototyping and future solution ideation. With this intent, the work met the definition of quality improvement outlined by NYU Grossman School of Medicine’s institutional review board and therefore was determined not to be human subjects research. Accordingly, neither ethics review nor consent was required. An institutional review board–approved quality improvement self-attestation was completed by the study team. Participants were informed of the project and its goals, and individual verbal consent was obtained at the onset of each activity.

## Results

### Participants

Shadowing was performed with three RNs, including 1 assistant nurse manager in an Observation unit and 2 staff nurses (1 in a telemetry unit and the other in a pediatric cardiac intensive care unit), each from 3 different hospitals. In-depth interviews were performed with a total of 7 participants, including 6 RNs and 1 PUA. Participants were from different units across 4 different hospitals. Interviews were conducted either in person or online via the online videoconferencing platform Webex (Cisco Systems). FGs were performed with 16 RN participants from 2 units in 2 different hospitals, with 1 participant being a nurse manager.

### Themes

Through the analysis of the interviews and FGs, 4 major themes emerged to characterize the participants’ perceptions of what the technology in an ideal inpatient room of the future would do and address. These themes include seamlessly integrated technologies, reducing the number of mental resources needed to complete a task (ie, cognitive load), communication improvement, and improvements for staff well-being.

#### A Need for Seamlessly Integrated Technologies

Many participants (n=8) emphasized the need for technologies to seamlessly work together to support quality of patient care, improve clinical staff workflow, and make tasks more efficient to reduce time and effort for task completion. Nurses (n=2) mentioned the burden of signing into and switching back and forth between multiple systems, indicating that to accomplish their work they are “required to switch between different systems and devices” [SN1] and noted that they “spend a good amount of time at the beginning of [their] shift signing into multiple systems” [SN1]. As a possible solution, one nurse said they “would love a centralized software that connects all the devices to integrate to all platforms” [FG1]. Multiple (n=6) participants noted that the systems and devices they use do not communicate with each other, increasing the work burden. In particular, nurses stated that tasks such as recording certain vitals relied on manual data entry, requiring the nurse to directly input measurements into the EHR [IN1]. To address this issue, one nurse stated they “would love more seamless integrations between systems to automate the flow of data without manual entry” [FG6]. Such as with “a device that the RN can use and information can flow into the chart wherever it is needed, reducing the number of clicks” [FG8]. Relatedly, the desire was expressed to “limit where documentation happens to fewer places…” [FG2]. In particular, it was suggested that “medication requests should be more seamless” [SN2] and that the current technologies “require nurses to use multiple systems and devices [resulting] in longer time performing the task and multiple trips back and forth” [SN2]. Furthermore, one nurse noted that systems not being able to communicate leads to an issue where “alarms continue to go off even when [they] are already at bedside caring for the patients because [the nurse’s] hands are too busy at the moment to shut the alarm off” [IN4].

#### Potential for Technology to Reduce Cognitive Load

A number of participants (n=12) highlighted how technologies that assist with the consolidation and prioritization of tasks could act as a personal assistant to help reduce the cognitive load placed on busy clinical staff. Participants emphasized that “nursing is one of the most interrupted jobs” [IN6] with many competing priorities, time constraints, and a need to multitask, sometimes leaving nurses saying, “I don’t know what to do next” [SN1]. One participant mentioned that they “would love to differentiate between critical needs and things that can wait” [FG4] and that “for task management and prioritization, [they] would love a running [list] of prioritized tasks for each patient so [they] can prioritize bathroom needs over water ask” [FG2] or a “program that would help prioritize alerts or silence alerts when [already being addressed]” [FG9]. Additional requests included, “a solution that can evaluate and differentiate… patient care needs [as they] relate to acuity, group proximity, efficiency” [FG14], “a flag for time sensitivity” [FG6], and something to “silence clinical equipment alerts if they are not real alerts” [FG12].

To handle constant interruptions, participants stated that they “would love a way to be reminded of tasks [they] haven’t completed, especially if time sensitive” [FG6]. Similarly, the participants expressed an interest in “reminders for common time-based tasks” [FG15], and the ability to “group low priority alerts that remind me every 20 min before another” [FG3]. One participant suggested that these alerts could come as “subtle audio or visual reminders of which tasks need to be accomplished at that time” [FG11]. To address the issue of prioritization and reminders, participants highlighted that they “would love a single mobile tech that is a single source of truth” [FG13], such as “an iPad with a list of priorities for nurses that minimize alert fatigue” [FG14].

#### Technology Enhancement of Interpersonal Communication

Some participants (n=5) saw an opportunity to simplify routine communication tasks with technologies that are user-friendly, streamline workflows, and ensure access to necessary information. The nurses (n=4) expressed challenges of communicating between themselves, with physicians, and with patients that may be remedied with improved technology. A potential opportunity area for technology to improve communication is during the handoff of a patient’s care. One nurse said that “For transfer of care from one nurse to the next, I would love to see a quick/brief overview of this patient’s current situation and needs so that I can quickly orient myself to what needs to be done” [IN1]. With another expressing the desire for a means of “writing things down quickly [such as with an] old whiteboard…for others to review without logging into Epic” [FG14]. A system to track task transfer was also desired, where the nurse “would love to submit a request for a nurse to assist with a specific task and be informed when it is complete” [SN2]. Emphasizing the need for this alternate communication, nurses expressed the need for “quicker communication without needing to be on Epic,” with one nurse reporting they “keep a small note pad in [their] badge with certain standard information for quick reference, such as phone numbers of physicians, because looking these up in the system is complex and time-consuming” [IN2].

To enhance communication technologies, some nurses (n=2) expressed a preference for hands-free communication options “to hear notifications in my ear (privately) and be able to respond hands-free” [IN4], such as an “ear piece a RN can wear” to get or “delay notifications until a specific time” [IN4] or be able to address “a call bell from another room or on my phone… to tell them ‘I’ll be there in a minute’” [FG10].

#### Opportunities for Improvement of Staff Well-Being

Several participants (n=4) mentioned how a clinician’s typical day can be physically demanding and how technology that reduces the physical burden of daily tasks would be beneficial. Within the theme of physical demands, subthemes arose, including reducing alarm sounds, patient movement, and supply management. Alarms represent a notable burden on staff, with one participant saying of the persistent sound, “I go home and still hear alarms” (shadowing PUA1). Participants expressed that they “would love the ability to not receive [alerts] when I am [already] in the room” [FG14] or to “turn off alerts for other patients while in [another] room with a patient” [FG15]. Clinicians also had concerns about the impact of auditory alarms on patients, expressing that, “for in-room alarms, I would love an option to change the parameters or silence in-room alarms, so that patients can sleep” [FG16].

Opportunities to relieve the physical burden of moving patients were highlighted as an important means of improving clinician well-being (n=4). Participants expressed the desire for “a device that would help move the patient easily, efficiently” [FG1] and for “help that doesn’t require another nurse” [FG7]. Suggestions were made for “the bed to do it or a way for a patient to do on their own” [FG6], such as a “robot or way to ask for a second person to come help” [FG2]. To improve “device management, moving patients, [and] excess alarms,” one participant indicated that they “would love wireless EKG leads, telemetry” [FG2].

The physical and time burden of supply and equipment management was expressed as a concern for participants (n=3). Participants expressed that “moving clinical devices, such as IV poles, Workstations on Wheels, from one room to another takes physical work and time” [SN2]. They wished for a way for the room to be “prepared with all the equipment and supplies I need to take care of my patients, ahead of time” [FG14] or an “easy way to find technologies needed at the bedside or…on the floor.” Medication administration was identified as another cumbersome routine task, requiring nurses to “make multiple trips back and forth between a patient room and medication dispensing system/cabinet to keep track whether a medication in need has been filled by the pharmacy” [SN2]. To solve these issues, one participant noted that they “would love a machine that delivers supplies when I need them for a patient at the bedside” [FG2], and “for the delivery of nonclinical, nonurgent things (blanket, pillows, water, linens, wheelchairs, and walkers), I would love the things to be delivered ad hoc that removes this burden on clinical staff so that they can focus on patient care.”

#### Envisioned Future Solutions

Table 1 describes future room concepts that emerged from qualitative work as they relate to these themes. Future solutions were envisioned to enhance patient monitoring and observation by using automated measurements and actions through computer vision and data triangulation. During the backcasting stage of analysis, a staff smart assistant via personal tech was envisioned, a device capable of real-time patient monitoring and escalation, task prioritization across patients, hands-free clinical documentation and communication, and with concise and actionable notifications. A smart supply management system integrated with the EHR was envisioned, capable of using computer vision to detect supply shortages and auto-deliver needed supplies.

**Table 1:** Mapping developed themes to envision future concepts for the “Inpatient Room of the Future.”

Theme	Future room concepts	
A need for seamlessly integrated technologies	Centralized software to connect and integrate all platforms Device that allows information to flow into the chart wherever it is needed Limit documentation to fewer places Computer vision and smart alarms that silence when the nurse is at the bedside Automated measurements and actions through computer vision Smart supply management system integrated with the EHR^a^ to ensure efficient coordination of supplies System that surfaces meaningful insights to clinical staff through data triangulation	
Potential for technology to reduce cognitive load	Software to assist with the consolidation and prioritization of tasks Software to differentiate patient care needs as they relate to acuity, group proximity, and efficiency AI^b^-enabled software assistant to reduce cognitive load Software to silence alerts already addressed Software that times less urgent alerts to occur after the nurse leaves the patient’s bedside Alerts with improved sensitivity to eliminate or silence clinical equipment alerts that are not true alerts Reminders for common time-based tasks that come as subtle audio or visual reminders Staff smart assistant via personal tech	
Technology enhancement of interpersonal communication	AI tools to provide a brief overview of the patient’s current situation and needs, so oncoming nurse can quickly orient to what needs to be done System to track task transfer to another nurse and be informed when complete Communication tool that does not require logging into the EHR Hands-free communication devices such as earpieces Ability to address call bells from another room using a phone	
Opportunities for improvement of staff well-being	Reducing alarm sounds Ability to turn off alarms for another patient in another room Ability to silence in-room alarms so patients can sleep Device to help move the patient easily that does not require another nurse Wireless ECG^c^ leads Smart supply system that delivers needed equipment, medications, and nonurgent things, such as blankets, to the patient's room	

^a^EHR: electronic health record.

^b^AI: artificial intelligence.

^c^ECG: electrocardiogram.

## Discussion

### Principal Findings

In this study, we found that inpatient nurses regularly interact with technology throughout their daily workflow [30]. Future technological improvements could reduce cognitive load, enhance communication, and promote staff well-being. Nurses showed interest in tools to reduce cognitive burden, including task consolidation aids to help manage prioritization amid frequent interprofessional communications and patient requests. Physical well-being was another significant theme with nurses' interest in technologies that could alleviate the physical effort involved with preparing rooms and moving patients. Future methods prepare organizations for technological breakthroughs by addressing pain points and possibilities [21].

### Situating Findings Within the Broader Context of Technology, Built Environment, and Nursing Futures

Over half of the participants noted that some technologies lacked the necessary integration with others. This finding is consistent with previous survey research findings that systems are not integrated with clinical workflows, creating an experience of many stand-alone solutions [31]. The integration of technology systems can play an important role in care quality and work satisfaction among nursing staff [32,33]. Successful health care technology integration requires a deliberate strategy [34]. For this reason, as near-term initiatives are considered to address needs, it is important to consider how the integration of new and existing technologies can align with the desired future vision of an inpatient room. Future concepts that emerged from our work included computer vision–enhanced patient monitoring of fall risk and wound healing, and data triangulation to provide meaningful insights to clinical staff.

Nursing staff expressed particular interest in technology to reduce the cognitive burden associated with managing multiple patients and frequent interruptions. Nurses indicated a need for more convenient communication methods and showed interest in informal mechanisms for interacting with patients and staff. As observed in previous research [12], participants emphasized the challenges of managing tasks amid frequent workflow disruptions and interprofessional communications. The introduction of technology-based cognitive support tools may positively affect clinical decision-making by supporting nurses’ decisions, thinking, and workflow [35]. Participants emphasized a desire to have a personal assistant–like tool to consolidate and prioritize tasks, reducing cognitive load. A smart staff assistant via personal tech may accomplish this. Emerging technologies such as artificial intelligence (AI) and machine learning may address these needs, with AI overlaying the integrated system to provide a higher level of care coordination. However, future research is needed to evaluate how these supportive technologies can be implemented without interfering with clinician judgment and critical thinking. Simulation studies with cognitive support tools could help identify potential issues with tool usability and its effect on clinician judgment under stress.

Participants raised a desire for technological solutions to improve physical well-being. Consistent with other settings, nurses emphasized the time and physical effort required to find necessary equipment for room preparation, medication administration, and movement of patients [36] and indicated an interest in solutions such as robotics [37]. Exploration of the use of delivery robots in inpatient care has produced mixed results, as it can lead to interruptions and possibly lead to a transfer of additional activities to the nurse [16]. Robotic assistive devices could help move patients and prevent back injuries [38-40]. Assistive robotics systems are mainly in the development and testing phases, and more research is necessary to ensure seamless integration with nursing workflows [16]. Previous research found concerns around job replacement due to robotics and the need for regulatory policies around the delegation of nursing tasks from humans to robots [41-43]. Innovations that reduce the physical and noise intrusiveness of monitoring devices [44-46], or change how materials and rooms are prepared before clinician involvement [44-46] may represent alternative solutions that can better support staff well-being. Further research is needed to identify the most beneficial types of technology.

Emerging technologies such as generative AI for communication and clinical decision support [47,48] and computer vision for video monitoring [49-51] are likely to have a function in inpatient settings [52]. To promote equity, future research is needed to assess potential differences in technological and training needs between subgroups of nurses, such as by age, and efforts should be made to co-design a future inpatient room vision with both technology partners and end users. Similarly, equity should be considered for other end users, including patients. Nurses must ensure that compassion and humanity remain central to patient care amidst technological advancements [53].

### Policy Recommendations and Other Considerations

To ensure new technologies and built environments align with clinical workflows, health care systems must prioritize interdisciplinary collaboration involving nurses, other clinical staff, technology developers, and facility planners from the early design stages. Adopting HCD principles in technology integration and health care facility design will help ensure innovations complement, rather than disrupt, care delivery. Future-facing approaches in co-designing physical environments (including virtual reality) have shown promise in non–health care settings and should be adapted to the health care space [54-56].

Flexibility and adaptability of infrastructure must be a priority, allowing for easy integration of future technologies while adapting to changing clinical needs. Cost-effective, long-term infrastructure investment is essential, focusing on modular designs for easy updates to physical hardware and technology. Green and sustainable design principles should be incorporated into building plans, promoting environmentally friendly practices, and reducing long-term operational costs. Health care systems must stay informed on the latest evidence regarding the impact of physical environments on patient outcomes and update infrastructure to optimize outcomes and reduce medical errors. Reforms in zoning, permitting, health and safety codes, and environmental and efficiency standards are essential for resilient, “future-proofed” building design [57].

To establish safe integration of emerging technologies, particularly AI-enabled features in nursing care, oversight mechanisms are imperative. The Food and Drug Administration and other safety oversight bodies should play a role in the evaluation and approval of AI-enabled devices to ensure trustworthy tools [58]. The European Artificial Intelligence Board, established under the AI Act [59], provides a model for coordinating AI policy. Privacy and security are key, and policies must safeguard patient information in line with guidelines from organizations such as the American Nurses Association [60]. Nurses should be actively involved at every stage of nursing-oriented technology development and implementation. Continuous education and training for nursing staff on emerging technologies and AI are essential to bridge the digital divide and ensure that nurses are equipped to use these tools effectively.

### Limitations

This study had several limitations. Foremost is the limited sample size prescribed by the operational nature of this study, and the inclusion of participants spanning multiple hospitals and units prevents the ability to test deductive hypotheses and perform subgroup analyses. The sampling strategy and sample size used may limit the generalizability of these results, particularly across diverse inpatient settings. Due to the small sample size, it is unclear whether thematic saturation was achieved for the various unit settings. Additionally, the use of a convenience sample may have led to selection bias, as those who volunteered for the study may have a different work environment experience than those not readily available for participation. Future research should consider expanding the scope to assess the experiences of a larger sample of staff types. Finally, as a precaution to protect patient and clinician privacy, the nurse interviews and FGs were not audio recorded. The interviews and FGs that occurred both in the hospital and office settings were captured via the field researcher’s written notes. While interviewer training protocols and note-taking templates were used to mitigate bias, a lack of audio recordings may have led to a loss of information or the introduction of bias from the notetakers. Additionally, due to the small sample size and the categorization of employees as a vulnerable group, the efforts to protect the confidentiality of participants limited the ability to collect demographic information. Thus, it limits the ability to analyze the impact of individual characteristics on outcomes and generalizability.

### Conclusion

As patient care continues to evolve and the diversity of available health technologies expands, nursing efficiency and care quality benefit from a medical future-focused approach to inform the design vision for an inpatient room of the future*.* The findings reveal that while current technologies address specific tasks, there are significant opportunities for technology integration, reducing cognitive load, enhancing communication, and promoting the physical and mental well-being of nursing staff. By applying futures methods, health care organizations can proactively identify and address these needs, so that advancements are aligned with the practical realities of nursing practice. RNs working in the inpatient setting highlight the importance of seamless integration of technologies to their work. Future research should focus on implementing supportive technologies that do not interfere with clinician judgment and critical thinking. Policy recommendations include oversight mechanisms for evaluating AI-enabled devices, safeguarding patient information, and ensuring nurses are actively involved at every stage of technology development and implementation. It is important to strive toward a deliberate and holistic vision for an inpatient room of the future. Future inpatient unit designs should actively engage input from both nursing professionals and technologists to ensure the development of integrated systems that deliver a harmonious user experience.

## Abbreviations

AI: artificial intelligence
EHR: electronic health record
FG: focus group
HCD: human-centered design
IN: interview nurse
PUA: Patient Unit Associate
RN: registered nurse
SN: shadowing nurse

## References

[1] Hua Y, Becker F, Wurmser T, Bliss-Holtz J, Hedges C (2012). Effects of nursing unit spatial layout on nursing team communication patterns, quality of care, and patient safety. HERD.

[2] MacAllister L, Zimring C, Ryherd E (2019). Exploring the relationships between patient room layout and patient satisfaction. HERD.

[3] Molin J, Strömbäck M, Lundström M, Lindgren B (2021). It's not just in the walls: patient and staff experiences of a new spatial design for psychiatric inpatient care. Issues Ment Health Nurs.

[4] Devlin AS, Andrade CC, Carvalho D (2016). Qualities of inpatient hospital rooms: patients' perspectives. HERD.

[5] Bayramzadeh S, Aghaei P (2021). Technology integration in complex healthcare environments: a systematic literature review. Appl Ergon.

[6] Cvach MM, Currie A, Sapirstein A, Doyle PA, Pronovost P (2013). Managing clinical alarms: using data to drive change. Nurs Manage.

[7] Brown KK, Gallant D (2006). Impacting patient outcomes through design: acuity adaptable care/universal room design. Crit Care Nurs Q.

[8] Reiling J, Hughes RG, Murphy MR, Hughes RG (2008). The impact of facility design on patient safety. Patient Safety and Quality: An Evidence-Based Handbook for Nurses.

[9] Mead M, Ibrahim AM (2022). Evaluating mortality and hospital room design after high-risk inpatient surgery. J Am Coll Surg.

[10] Umansky J, Rantanen E (2016). Workload in nursing. Proc Hum Factors Ergon Soc Annu Meet.

[11] Sun C, Fu C, Cato K (2024). Characterizing nursing time with patients using computer vision. J Nurs Scholarsh.

[12] Panattoni N, Sperduti I, Spano A, De Leo A, Petrone F, Di Simone E (2024). Care call requests and inpatient beds modernization: is there any link? A prospective observational study in the oncological setting. J Adv Nurs.

[13] Michel O, Garcia Manjon AJ, Pasquier J, Ortoleva Bucher C (2021). How do nurses spend their time? A time and motion analysis of nursing activities in an internal medicine unit. J Adv Nurs.

[14] Ruppel H, Funk M (2018). Nurse-technology interactions and patient safety. Crit Care Nurs Clin North Am.

[15] Forde-Johnston C, Butcher D, Aveyard H (2023). An integrative review exploring the impact of electronic health records (EHR) on the quality of nurse-patient interactions and communication. J Adv Nurs.

[16] Ohneberg C, Stöbich N, Warmbein A, Rathgeber I, Mehler-Klamt AC, Fischer U, Eberl I (2023). Assistive robotic systems in nursing care: a scoping review. BMC Nurs.

[17] Greer V, Johnson E, Hsu J (2021). Variables and outcomes in patient room design: a study of design hypotheses. HERD.

[18] Dawson J, Phanich KJ, Wiese J (2024). Reenvisioning patient education with smart hospital patient rooms. Proc ACM Interact Mob Wearable Ubiquitous Technol.

[19] Lunenfeld P (2003). Design Research: Methods and Perspectives.

[20] Hassenzahl M (2014). User experience and experience design. The Encyclopedia of Human-Computer Interaction, 2nd Ed.

[21] Meskó B, Kristóf T, Dhunnoo P, Árvai N, Katonai G (2024). Exploring the need for medical futures studies: insights from a scoping review of health care foresight. J Med Internet Res.

[22] Vredenburg K, Isensee S (2001). User-Centered Design: An Integrated Approach. User-Centered Design: An Integrated Approach.

[23] What is design thinking (DT). Interaction Design Foundation.

[24] Candy S, Kornet K (2019). Turning foresight inside out: an introduction to ethnographic experiential futures. J Futures Stud.

[25] Evans M (2010). Design futures: an investigation into the role of futures thinking in design.

[26] Bezold C (1995). The future of health futures. Futures.

[27] Figma.

[28] Spencer L, Ritchie J (2002). Qualitative data analysis for applied policy research. Analyzing Qualitative Data.

[29] Strauss A, Corbin J (1990). Basics of qualitative research: Grounded theory procedures and techniques.

[30] Garcia Gonzalez-Moral S, Beyer FR, Oyewole AO, Richmond C, Wainwright L, Craig D (2023). Looking at the fringes of MedTech innovation: a mapping review of horizon scanning and foresight methods. BMJ Open.

[31] Topaz M, Ronquillo C, Peltonen LM, Pruinelli L, Sarmiento RF, Badger MK, Ali S, Lewis A, Georgsson M, Jeon E, Tayaben JL, Kuo CH, Islam T, Sommer J, Jung H, Eler GJ, Alhuwail D, Lee YL (2017). Nurse informaticians report low satisfaction and multi-level concerns with electronic health records: results from an international survey. AMIA Annu Symp Proc.

[32] Alshammari MH, Alenezi A (2023). Nursing workforce competencies and job satisfaction: the role of technology integration, self-efficacy, social support, and prior experience. BMC Nurs.

[33] Zadvinskis IM, Garvey Smith J, Yen PY (2018). Nurses' experience with health information technology: longitudinal qualitative study. JMIR Med Inform.

[34] Schoville RR, Titler MG (2015). Guiding healthcare technology implementation: a new integrated technology implementation model. Comput Inform Nurs.

[35] Harmon CS, Adams S, Davis JE (2020). Nursing cognitive-overload and electronic documentation burden: a literature review. J Inform Nurs.

[36] Nazarian M, Price A, Demian P, Malekzadeh M (2018). Design lessons from the analysis of nurse journeys in a hospital ward. HERD.

[37] Madi M, Nielsen S, Schweitzer M, Siebert M, Körner D, Langensiepen S, Stephan A, Meyer G (2024). Acceptance of a robotic system for nursing care: a cross-sectional survey with professional nurses, care recipients and relatives. BMC Nurs.

[38] Kato K, Yoshimi T, Tsuchimoto S, Mizuguchi N, Aimoto K, Itoh N, Kondo I (2021). Identification of care tasks for the use of wearable transfer support robots - an observational study at nursing facilities using robots on a daily basis. BMC Health Serv Res.

[39] Georgadarellis GL, Cobb T, Vital CJ, Sup FC (2024). Nursing perceptions of robotic technology in healthcare: a pretest-posttest survey analysis using an educational video. IISE Trans Occup Ergon Hum Factors.

[40] Davis JH (2024). Leading Regulatory Policy: RN Delegation to AI Humanoid Robots. CIN: Computers, Informatics, Nursing.

[41] Wong DLT, Yu J, Li Y, Deepu CJ, Ngo DH, Zhou C, Singh SR, Koh A, Hong R, Veeravalli B, Motani M, Chua KC, Lian Y, Heng CH (2020). An integrated wearable wireless vital signs biosensor for continuous inpatient monitoring. IEEE Sensors J.

[42] Poncette AS, Spies C, Mosch L, Schieler M, Weber-Carstens S, Krampe H, Balzer F (2019). Clinical requirements of future patient monitoring in the intensive care unit: qualitative study. JMIR Med Inform.

[43] Darbyshire JL, Müller-Trapet M, Cheer J, Fazi FM, Young JD (2019). Mapping sources of noise in an intensive care unit. Anaesthesia.

[44] Karapinar Y, Habib A, Sawyerr H (2017). Improving time efficiency gathering equipment in the treatment room. BMJ Open Qual.

[45] Gravina N, VanWagner M, Austin J (2008). Increasing physical therapy equipment preparation using task clarification, feedback and environmental manipulations. J Organ Behav.

[46] Tucker AL, Heisler WS, Janisse LD (2014). Designed for workarounds: a qualitative study of the causes of operational failures in hospitals. Perm J.

[47] Moulaei K, Yadegari A, Baharestani M, Farzanbakhsh S, Sabet B, Reza Afrash M (2024). Generative artificial intelligence in healthcare: a scoping review on benefits, challenges and applications. Int J Med Inform.

[48] Yim D, Khuntia J, Parameswaran V, Meyers A (2024). Preliminary evidence of the use of generative AI in health care clinical services: systematic narrative review. JMIR Med Inform.

[49] Jorge J, Villarroel M, Tomlinson H, Gibson O, Darbyshire JL, Ede J, Harford M, Young JD, Tarassenko L, Watkinson P (2022). Non-contact physiological monitoring of post-operative patients in the intensive care unit. NPJ Digit Med.

[50] Cournan M, Fusco-Gessick B, Wright L (2018). Improving patient safety through video monitoring. Rehabil Nurs.

[51] Lindroth H, Nalaie K, Raghu R, Ayala IN, Busch C, Bhattacharyya A, Moreno Franco P, Diedrich DA, Pickering BW, Herasevich V (2024). Applied artificial intelligence in healthcare: a review of computer vision technology application in hospital settings. J Imaging.

[52] Sensmeier J, Ivory CH (2018). Technology myth busters for nurse leaders. J Nurs Adm.

[53] Wiljer D, Charow R, Costin H, Sequeira L, Anderson M, Strudwick G, Tripp T, Crawford A (2019). Defining compassion in the digital health age: protocol for a scoping review. BMJ Open.

[54] Dane G, Evers S, van den Berg P, Klippel A, Verduijn T, Wallgrün JO, Arentze T (2024). Experiencing the future: evaluating a new framework for the participatory co-design of healthy public spaces using immersive virtual reality. Comput Environ Urban Syst.

[55] Paraschivoiu I, Dziabiola M, Meschtscherjakov A (2023). Postcards from the future: Speculating the future of built environments with citizens. https://dl.acm.org/doi/abs/10.1145/3593743.3593784.

[56] Vikström L, Ek K, Luciani A, Rizzo A (2025). Co-designing the urban energy transition: a resident-based approach. Cities.

[57] Watt H, Davison B, Hodgson P, Kitching C, Densley Tingley D (2023). What should an adaptable building look like?. Resour Conserv Recycl Adv.

[58] Warraich HJ, Tazbaz T, Califf RM (2025). FDA perspective on the regulation of artificial intelligence in health care and biomedicine. JAMA.

[59] AI Board. European Comission.

[60] Privacy and confidentiality. American Nurses Association (ANA).

